# Intention Retrieval and Deactivation Following an Acute Psychosocial Stressor

**DOI:** 10.1371/journal.pone.0085685

**Published:** 2013-12-27

**Authors:** Moritz Walser, Rico Fischer, Thomas Goschke, Clemens Kirschbaum, Franziska Plessow

**Affiliations:** Department of Psychology, Technische Universität Dresden, Dresden, Germany; University College London, United Kingdom

## Abstract

We often form intentions but have to postpone them until the appropriate situation for retrieval and execution has come, an ability also referred to as event-based prospective memory. After intention completion, our cognitive system has to deactivate no-more-relevant intention representations from memory to avoid interference with subsequent tasks. In everyday life, we frequently rely on these abilities also in stressful situations. Surprisingly, little is known about potential stress effects on these functions. Therefore, the present study aimed to examine the reliability of event-based prospective memory and of intention deactivation in conditions of acute psychosocial stress. To this aim, eighty-two participants underwent the Trier Social Stress Test, a standardized stress protocol, or a standardized control situation. Following this treatment, participants performed a computerized event-based prospective memory task with non-salient and focal prospective memory cues in order to assess prospective memory performance and deactivation of completed intentions. Although the stress group showed elevated levels of salivary cortisol as marker of a stress-related increase in hypothalamus-pituitary-adrenal axis activity throughout the cognitive testing period compared to the no-stress group, prospective memory performance and deactivation of completed intentions did not differ between groups. Findings indicate that cognitive control processes subserving intention retrieval and deactivation after completion may be mostly preserved even under conditions of acute stress.

## Introduction

We are often confronted with situations in which we have to postpone action execution to a later point in time. The ability to remember to perform an intended action after some delay in the future is known as prospective memory (PM), which requires the retrieval of an intended action in the absence of a direct instruction, either at a pre-specified point of time (i.e., time-based PM) or to the appearance of an external mnemonic cue (i.e., PM cue; event-based PM) [1]. In addition to that, intention representations need to be deactivated once the intended action has been completed in order to prevent interference of completed intention representations with subsequent task performance [2–4]. Given continuous increase of work intensity in modern society, for example, high working speed and tight deadlines [5] with general increased numbers of workflow interruptions [6], it is surprising that the reliability of PM functioning in everyday life of healthy subjects has only recently attracted research in the scientific community, for example, with respect to mood states [7] or sleep disturbances [8]. 

Yet, one of the most important factors affecting daily life performance is the experience of stress. Paralleling the expanding complexity and intensity of work life, stress has become omnipresent with considerable effects on physical and mental health. When being stressed, the human body responds on two different physiological stress axes, namely a rapid increase of sympathetic nervous-system (SNS) activity and a slower increase of hypothalamus-pituitary-adrenal (HPA) axis activity. More precisely, stress-induced increased SNS activity is associated with increased catecholamine release which decreases firing of prefrontal cortex (PFC) neurons [9,10]. Increased HPA axis activation following an acute psychosocial stressor is associated with the synthesis and release of glucocorticoids (i.e., cortisol) into the bloodstream [11]. Glucocorticoids bind to glucocorticoid receptors that are widespread in the PFC [12,13] and alter PFC activity [14,15]. This evidence that both stress axes have the potential to strongly affect PFC functioning [16], provides a potential physiological link of how acute stress might interact with PM performance and intention deactivation, which have also been closely related to PFC functioning [17,18].

To the best of our knowledge, there is only a single study by Nater and colleagues that followed this assumption and experimentally manipulated stress to examine its role on PM performance [19]. Participants performed a word rating task as ongoing task with an embedded PM task which required them to either press a target key upon the onset of rarely occurring PM cue words (event-based PM) or to press a target key every two minutes (time-based PM). In the time-based condition, participants had the option to check on a clock display for better time monitoring (clock checks), that is, a specific key press presented a clock for 2 sec on the monitor. Importantly, for time-based PM, quality of PM performance was *higher* following stress induction compared to a non-standardized resting condition. This apparent “improvement” of time-based PM performance following stress, however, was most likely not related to enhanced memory functioning. Instead, stressed individuals increased the number of self-induced clock checks which naturally improves time monitoring. These results are therefore in line with other demonstrations of an induced shift of processing strategy as a consequence of experienced stress [20,21]. 

In the Nater et al. study such trade-offs in processing strategy were only observed for time-based PM but not for event-based PM (i.e., no effects of stress on event-based PM hit rates were observed), which the authors explained by time-based PM being more resource-demanding compared to event-based PM [19]. At the same time, hints for potential shifts of processing strategies under stress for event-based PM might have been difficult to detect as, for example, PM performance quality was exclusively based on accuracy measures whereas potential sacrifices in PM response duration were not taken into account. Importantly, it is conceivable that stress induction might have also affected initiation of PM task performance as reflected in response times (RTs; for an example of effects on PM trial RTs but not error rates see 22). Further potential alterations in processing strategies might be related to trade-offs in the performance of the two tasks, that is, ongoing and PM task, respectively. In a recent dual-task study in our lab, we could show that an acute stress experience leads to a change in dual-task processing strategy towards more resource-saving integrative and parallel task processing mode compared to a more resource-demanding distinct and serial dual-task processing mode in the no-stress control condition [21]. These findings highlight the necessity of reporting performance measures of both tasks in order to detect potential between-task processing trade-offs. Unfortunately, Nater et al. [19] did not report ongoing-task performance. Therefore, it remains an open albeit theoretically important question whether similar trade-offs between tasks may be found in event-based PM under the influence of stress. 

**Figure 1:**
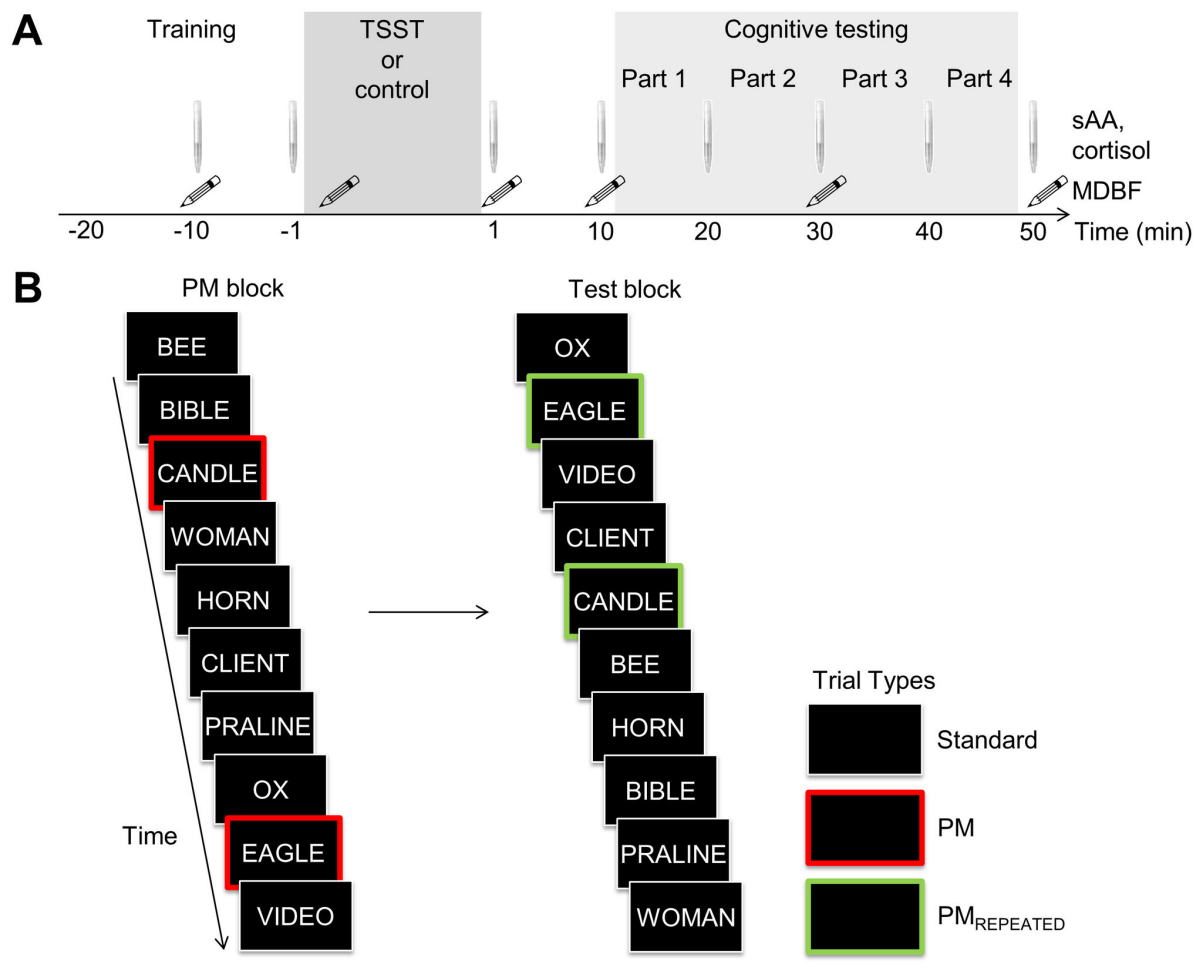
Procedure. (A) Schematic illustration of the procedure with the components training of the cognitive task, treatment (i.e., Trier Social Stress Test, TSST, or standardized control situation) and cognitive testing (including testing parts 1-4). Note that each part comprised two PM block-Test block cycles. In addition, measurement time-points of salivary α-amylase (sAA), cortisol and mental-state with the German “Mehrdimensionaler Befindlichkeitsfragebogen” (multidimensional mental-state questionnaire, MDBF [31]) are given. Note that at time point -1 min, the saliva sample was taken before TSST or control treatment, whereas the MDBF was completed after treatment instruction, to enable assessing anticipation of the upcoming treatment. (B) Example trial sequence of the prospective memory (PM) block and Test block. As ongoing task participants performed animate vs. inanimate categorizations on German nouns in all trials except for PM trials, on which they were required to press the spacebar. Aftereffects of completed intentions were assessed in the Test block as ongoing-task performance differences between PM_REPEATED_ compared to standard trials. Note colored framing of trial types was not present in the experiment but serves exclusively to illustrate different trial types in this figure.

Therefore, in the present study we set out to extend the previous study by Nater et al. [19] by providing a more detailed test of acute psychosocial stress potentially affecting PM performance and intention deactivation, taking the possibility of processing strategy trade-offs into account. For this, we adopted a version of the PM paradigm [2,3], which consisted of a PM block to measure PM performance and a Test block to assess aftereffects of completed intentions as indicator for intention-deactivation ability (Figure 1). As ongoing task participants categorized words as animate or inanimate. In the PM block, an additional PM task required participants to monitor for specific PM cue words (e.g., *eagle* and *candle*) and to respond with a pre-specified PM key. At the end of the PM block participants were informed that the PM task was completed. No-longer-relevant PM cue words (i.e., PM_REPEATED_ trials) were re-presented in the Test block without a specific instruction attached to them. Aftereffects of completed intentions were measured as performance differences between PM_REPEATED_ trials and ongoing-task standard trials during the Test block. 

In order to enable detecting possible stress effects, we developed a paradigm supporting resource-consuming monitoring-based retrieval during the PM block as well as reliable aftereffects of completed intentions during the Test block. Given that monitoring is rather supported by non-focal and non-salient PM cues [23,24] whereas aftereffects could at least be partly explained by spontaneous retrieval [2,3,18] which is supported by focal and salient cues, we tried to find a compromise between task characteristics supporting monitoring versus spontaneous retrieval by using focal and non-salient PM/PM_REPEATED_ cues (similar to [19]). In the present study, the term focal denotes that PM cue information was also decoded during ongoing-task processing because both tasks strongly overlapped (e.g., the semantic of the PM cue word *candle* needed to be processed at the same time for the ongoing animate/inanimate categorization). In contrast, in non-focal PM tasks PM cues demand additional processing as required for the ongoing task (e.g., when PM cues are all words containing the syllable *can*, e.g., *candle, applicant, hurricane*) [25]. In the present study non-salient refers to the fact that PM cues did not clearly perceptually deviate from standard trials as opposed to e.g., salient PM cues written in red font color.

### Stress was induced by exposing half of the participants to the Trier Social Stress Test

(TSST) [26], a standardized stress protocol considered to be the best tool for stress induction with the largest cortisol and adrenocorticotropin hormone changes and the longest times to recovery compared to other stress induction techniques [27]. The other half of the participants underwent a standardized control condition [28]. In order to allow attributing potential stress-induced alterations in cognitive performance, we assessed SNS and HPA axis activity by analyzing salivary α-amylase (sAA) and salivary cortisol, respectively [29,30]. In addition, we assessed subjective mood, arousal, and fatigue by the standardized questionnaire MDBF (*Mehrdimensionaler Befindlichkeitsfragebogen*, multidimensional mental-state questionnaire) [31].

 Following the assumption that stress generally impairs prefrontal-cortex processing [16], a straight-forward prediction is that PFC related higher-cognitive functions should suffer from an acute stress experience [32–34]. With respect to PM performance, this should be especially evident for the efficiency of PM-cue monitoring, the neural correlates of which have previously been related to the PFC [17,18]. Similarly, as the control of interference from re-activated formerly relevant but completed intention representations has also been linked to neural activations in the PFC [18], reduced PFC processing under stress should lead to increased aftereffects of completed intentions in the Test block. 

Following the assumption of resource re-allocation under stress as a compensation strategy for increased stress-induced demands [35,36], it is conceivable to obtain evidence of a shift in processing strategy [19] that might be associated with a parsimonious processing mode to save cognitive resources [21] and/or with increased individual task processing efficiency under stress [37]. For example, a stress-induced shift towards a more integrative holistic processing mode [21] might increase PM-performance quality at the cost of quality in ongoing-task performance. Alternatively, a stress-induced increase of ongoing-task processing efficiency [37] might release capacities for the PM task, resulting in increased PM performance quality. In any case, such forms of resource re-allocation should be observable in shifts of between-task processing that are detectable in trade-offs between ongoing-task processing and PM task processing. Finally, recent findings of increased shielding of task relevant processing from task-irrelevant information [20,35] predict smaller aftereffects of completed intentions following stress compared to no-stress conditions.

Because recent evidence suggests that stress effects can be gender-specific [38,39], we further extended the Nater et al. [19] study by specifically testing male and female participants that were equally distributed across both treatment groups. Finally, because it has been reported that stress might exert influences on memory and executive functions only during very specific and narrow time intervals, either directly following stress [38,40] or after an increasing time-lag after stressor cessation [20,41], we included the factor time in the analyses of cognitive performance. 

## Methods

### Participants

Eighty-two students of the Technische Universität Dresden (41 male; 18 - 29 years, *M* = 21.96 years, *SD* = 2.68 years; 72 right-handed) participated in a single 2 hours long experimental session taking place between noon and 8 pm. Testing took place in the afternoon to avoid effects of cortisol awakening response as well as most pronounced inter-individual variance in salivary-free cortisol as reported during morning times [42]. Volunteers received 12 € or course credits. All participants were healthy, medication free, of normal weight (body mass index between 18 and 27, *M* = 22.05, *SD* = 2.17) because stress reactivity might be affected by these aspect [43]. Participants had normal or corrected-to-normal vision. Given that reduced physiological stress responses were reported for habitual smokers [44] and oral-contraceptive intake [42], participants were non-smokers (i.e., less than 5 cigarettes per week) and female participants refrained from using hormone-based birth control. Note that we did not control for menstrual-cycle phase, because previous studies did not find an effect of menstrual-cycle phase [40] or found reliable and comparable stress reactivity in female compared to male participants when not controlling for this factor [21,41]. 

### Ethics Statement

The study was approved by the Institutional Review Board of the Technische Universität Dresden and conducted in accordance to ethical standards of the 1964 Declaration of Helsinki. All participants gave informed written consent to take part in the study.

### Stress Induction and Stress Validation

Participants were randomly assigned to a stress (20 male, 21 female) or a no-stress control group (21 male, 20 female). For stress induction, we used the TSST [26] which consists of an anticipatory period followed by public speaking and a mental arithmetic task in front of a committee and a video camera (total time: 15 min). For the no-stress group, we used a standardized control situation strongly matching the TSST without its stress-inducing features [28]. To measure biological stress levels, saliva samples were collected 10 and 1 min before treatment onset and 1, 10, 20, 30, 40, and 50 min after treatment cessation by means of salivette sampling devices (Sarstedt, Nümbrecht, Germany) (Figure 1). Note that between measurement time-points -1 min and 1 min passed 22 minutes because additionally to the 15 min stress/no-stress treatment, it took 5 min to go to and return from the treatment room. From saliva samples, salivary α-amylase (sAA) and salivary cortisol were analyzed by using a quantitative enzyme-kinetic method [45] and a chemiluminescence immunoassay (CLIA, IBL International, Hamburg, Germany), respectively. Intra- and interassay variabilities were less than 8%. Subjective individual stress levels were assessed with the MDBF mood questionnaire [31], administered six times throughout the session (10 and 1 min before and 1, 10, 30 and 50 min after treatment). Except for time point 1 min prior to TSST or control treatment, the MDBF was completed while providing the saliva samples. Importantly, at -1 min, a saliva sample was taken before treatment instruction (stress vs. no-stress protocol). The MDBF, however, was completed after treatment instruction to ensure that participants were aware of the upcoming treatment procedure.

### Cognitive Task

Target stimuli (words of animate vs. inanimate objects, visual angle of 0.9° × 3.5° at a viewing distance of 60 cm) were centrally displayed using a Windows XP SP2 personal computer running Presentation software (Version 0.71; www.neurobs.com) on a 17-inch monitor. Participants responded with the left (*S* key) and the right (*L* key) index finger on a QWERTZ keyboard to words of animate or inanimate objects (counterbalanced over participants), respectively. For PM trials participants pressed the spacebar with the thumb of their dominant hand.

Twenty-four trials of animate-inanimate categorization served as ongoing-task practice block. Each trial started with a fixation sign (plus sign, 400 ms). Then, the target word was shown until a response was given (2,000 ms max). If no or an erroneous response was provided, a high pitch tone (700 Hz) was presented for 150 ms as feedback through headphones. 

Subsequently and in contrast to the Nater et al. study [19], participants were engaged in extensive training for three cycles of a PM block and a Test block prior to the stress/no-stress treatment in order to prevent learning and practice effects [20,41]. Following treatment, the actual experiment consisted of eight cycles. Each cycle consisted of a PM block and a Test block, whereas the first served to assess PM performance and the latter to measure aftereffects of completed intentions. In the PM block, participants had to respond to two specific words (i.e., PM cues, e.g., *candle* and *eagle*) by pressing the spacebar instead of performing the animate-inanimate categorization ongoing task (Figure 1). In the Test block, participants performed the animate-inanimate categorization task on all trials. No-more-relevant PM cues from the PM block were re-presented as PM_REPEATED_ trials to test aftereffects of completed intentions. During each cycle, eleven animate and inanimate words were presented randomly four times each during the PM block and the Test block, respectively (total of 88 trials per block), whereas one animate and one inanimate word was randomly drawn to serve as PM/PM_REPEATED_ cue. For each participant, eleven out of 121 animate and inanimate words were randomly assigned to one of the eleven repeated cycles. Animate and inanimate words were matched concerning word length and initial letter.

### Procedure

At session start, written informed consent was obtained and basic demographic information was assessed. Then, participants trained the cognitive task before they underwent the stress or no-stress treatment at twenty minutes after the beginning of the experimental session. From 10 min to 50 min after stress cessation, the actual experimental cognitive task was administered. In order to reduce variations in glucose levels participants had to refrain from eating and drinking sugar-based drinks two hours before the experimental session. At session start all participants received 200 ml grape juice to standardize inter-individual glucose level, the availability of which is a prerequisite for the stress-induced increase of HPA-axis activity [46].

### Data Analysis

We computed mixed analyses of variance (ANOVAs) with the within-subjects factor time (eight or six levels, respectively) and the between-subjects factor treatment (stress vs. no stress) and sex (female vs. male) on logarithmized cortisol and sAA data [47], and total scores of the three MDBF dimensions in order to analyze changes in physiological and subjective stress levels over the time-course of the experimental session. Cognitive data (RTs and error rates) of PM and Test block after treatment entered mixed ANOVAs including the within-subjects factors trial type (PM block: standard vs. PM; Test block: standard vs. PM_REPEATED_) and time (first vs. second vs. third vs. fourth part), and the between-subjects factors treatment (stress vs. no stress) and sex (female vs. male). Note, each of the four parts comprised two cycles of PM and Test block. In contrast to previous studies from our lab [20,41], we used four instead of two parts to enable tracking possible time effects even more precisely. Furthermore, ongoing-task performance between PM and Test blocks was compared to assess potential monitoring costs in PM blocks that reflect a reliance on resource-demanding intention retrieval processes in PM performance [48] as opposed to rather spontaneous retrieval [49]. The ANOVAs contained the within-subject factors block (PM block vs. Test block) and time (first vs. second vs. third vs. fourth part) and the between-subject factors treatment (stress vs. no stress) and sex (female vs. male) on standard trial RTs and error rates.

For RT analyses, error trials (6.1%) and RTs differing more than 2.5 *SD*s from mean RT of each participant and condition (3.1%) were excluded. Because participants hardly made any commission errors (0.01%), these errors were not separately analyzed. 

## Results

### Stress Response

#### Biological measures

**Figure 2:**
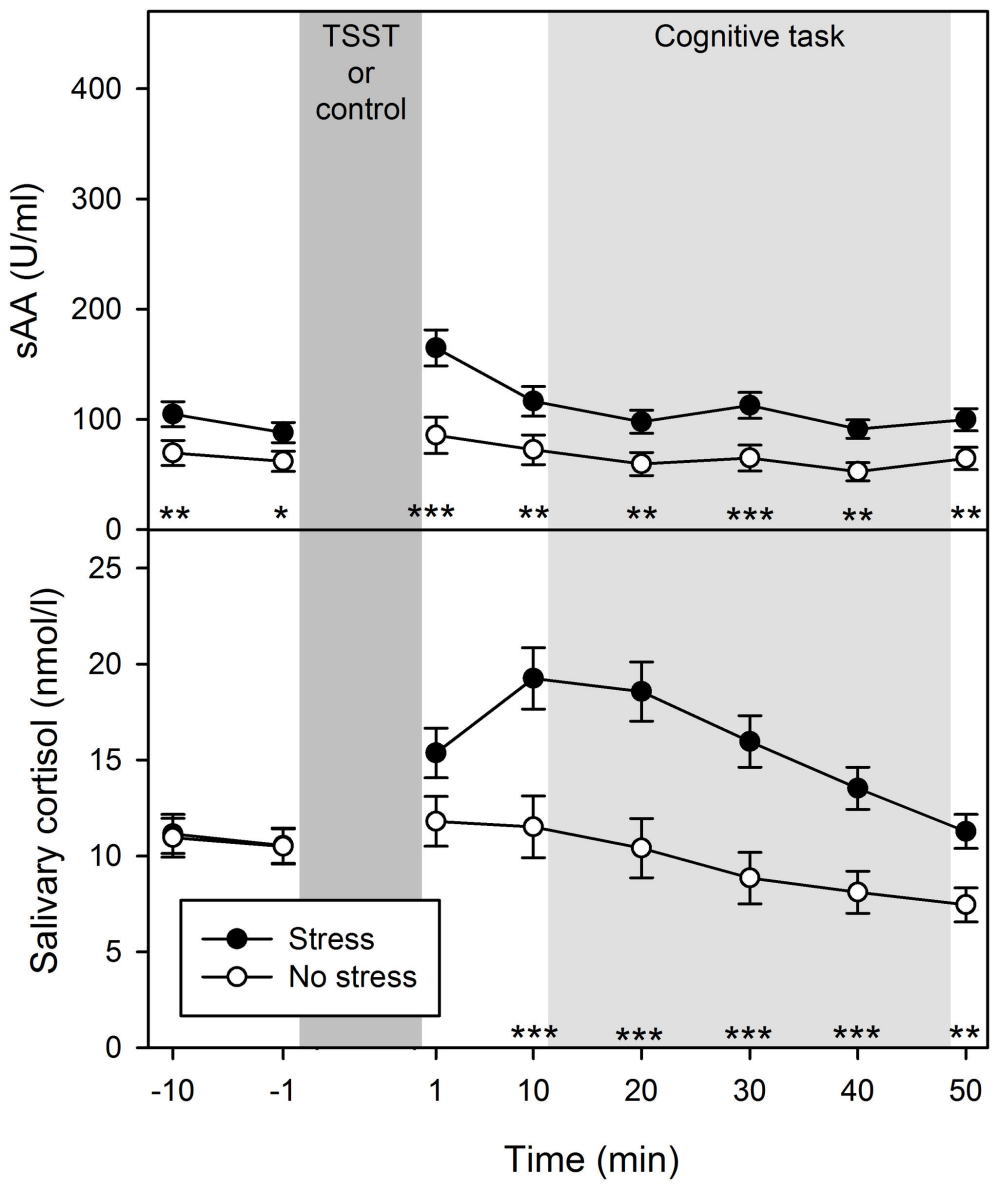
Neuroendocrine measures. Mean salivary α-amylase (sAA) and cortisol levels for the stress group and the no-stress group over the time-course of the experimental session (minutes before or after the Trier Social Stress Test [TSST] or the control condition, respectively). Error bars represent standard errors. *p < .05, **p < .01, ***p < .001.

Time course of cortisol levels differed between treatment groups, *F*(7, 546) = 11.66, *p* < .001, η^2^ = .13, with higher levels in the stress than no-stress group from 10 min after treatment as indicated by post-hoc *t* tests, ps ≤ .004 (other ps > .078) (Figure 2). Time and treatment revealed significant main effects, *F*(7, 546) = 20.16, *p* < .001, η^2^ = .21, and *F*(1, 78) = 7.61, *p* = .007, η^2^ = .09, respectively. Although main effects of time, *F*(7, 546) = 11.65, *p* < .001, η^2^ = .13, and treatment, *F*(1, 78) = 12.90, *p* = .001, η^2^ = .14, were also found for sAA, sAA levels were similarly increased in the stress group at all measurement time points, as time and treatment did not interact, *F*(7, 546) = 1.37, *p* = .242, η^2^ = .02. We neither observed sex effects nor any further significant effects for cortisol or sAA, ps > .112.

It has been shown that stress reactivity is comparable when the TSST is performed between the late morning and 19:00 h [50]. In order to avoid potential effects of measurement-time point on stress reactivity, in the present study testing deliberately started for half of the participants between 12:00 h and 15:00 h, and for the other half between 15:00 h and 18:00 h, whereas approximately the same amount of stress and no-stress participants were tested during both intervals. Importantly, measurement-time point did not affect cortisol nor sAA stress responses in either treatment group. This was indicated by similar areas under the curve with respect to increase [51] based on measurement-time point 1 min before treatment as baseline and all measurement-time points after stressor cessation (1, 10, 20, 30, 40, 50 min) for early versus late testing, ps > .393.

#### Mental state

**Figure 3:**
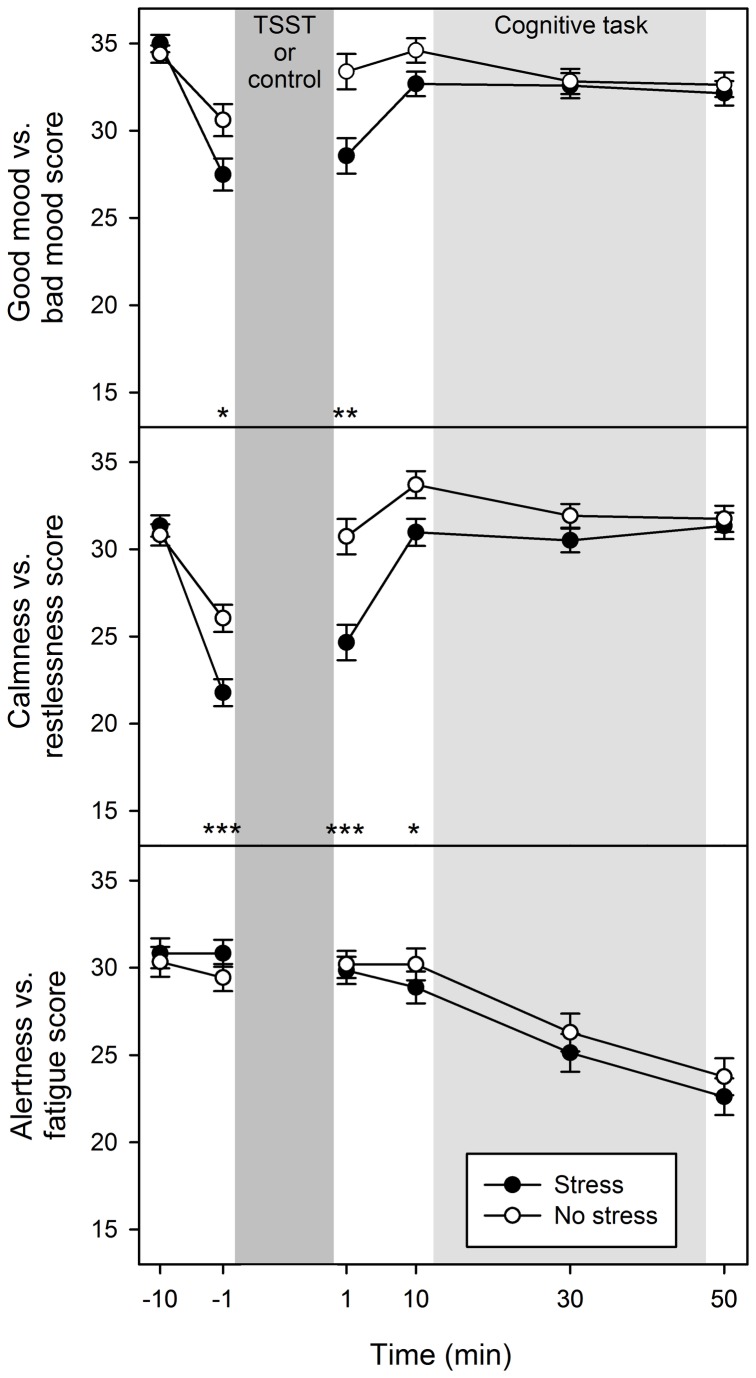
Mental state. Mean mental state scores on the three subscales good mood vs. bad mood, calmness vs. restlessness, alertness vs. fatigue from the German “Mehrdimensionaler Befindlichkeitsfragebogen” (multidimensional mental-state questionnaire, MDBF [31]) for the stress group and the no-stress group over the time-course of the experimental session (minutes before or after the Trier Social Stress Test [TSST] or the control condition, respectively). Error bars represent standard errors. *p < .05, **p < .01, ***p < .001.

Time courses of subjective mood varied between treatment groups, *F*(5, 390) = 10.31, *p* < .001, η^2^ = .12, with stressed participants reporting worse mood after they were instructed for the upcoming treatment (-1 min), *t*(80) = -2.40, *p* = .019, d = -0.53, and directly after treatment (1 min), *t*(80) = -3.37, *p* = .001, d = -0.75 (other ps > .055; Figure 3). Stress differentially affected mood in both sexes, as shown by a Treatment × Sex interaction, *F*(1, 78) = 4.86, *p* = .030, η^2^ = .06, and a Time × Treatment × Sex interaction, *F*(5, 390) = 2.50, *p* = .048, η^2^ = .03. Within females, mood was worse in the stress than no-stress group at -1 min, *t*(39) = -2.79, *p* = .008, d = -0.87, at 1 min, *t*(39) = -3.96, *p* < .001, d = -1.23, and at 10 min, *t*(39) = -2.32, *p* = .026, d = -0.73 (other ps > .144), as indicated by a Time × Treatment interaction, *F*(5, 195) = 9.69, *p* < .001, η^2^ = .20. In contrast, within males the time course of mood was similar between treatment groups, *F*(5, 195) = 2.10, *p* = .094, η^2^ = .05.

 Further, stressed participants felt more restless at -1 min, *t*(80) = -3.89, *p* < .001, d = -0.86, at 1 min, *t*(80) = -4.24, *p* < .001, d = -0.93, and at 10 min, *t*(80) = -2.50, *p* = .015, d = -0.55, as revealed by a Time × Treatment interaction, *F*(5, 390) = 10.17, *p* < .001, η^2^ = .11. The time course in restlessness versus calmness differed between sexes, *F*(5, 390) = 3.70, *p* = .009, η^2^ = .05, with females tending to be more restless than males at 1 min, *t*(80) = -1.84, *p* = .070, d = -0.41 (other ps > .155). 

 Fatigue increased over time, *F*(5, 390) = 55.38, *p* < .001, η^2^ = .42, with similar mean fatigue levels, *F* < 1, and a similar time course in treatment groups, *F*(5, 390) = 1.72, *p* = .155, η^2^ = .02, and no sex effects, ps > .085. 

### Cognitive Performance

#### PM block

**Figure 4:**
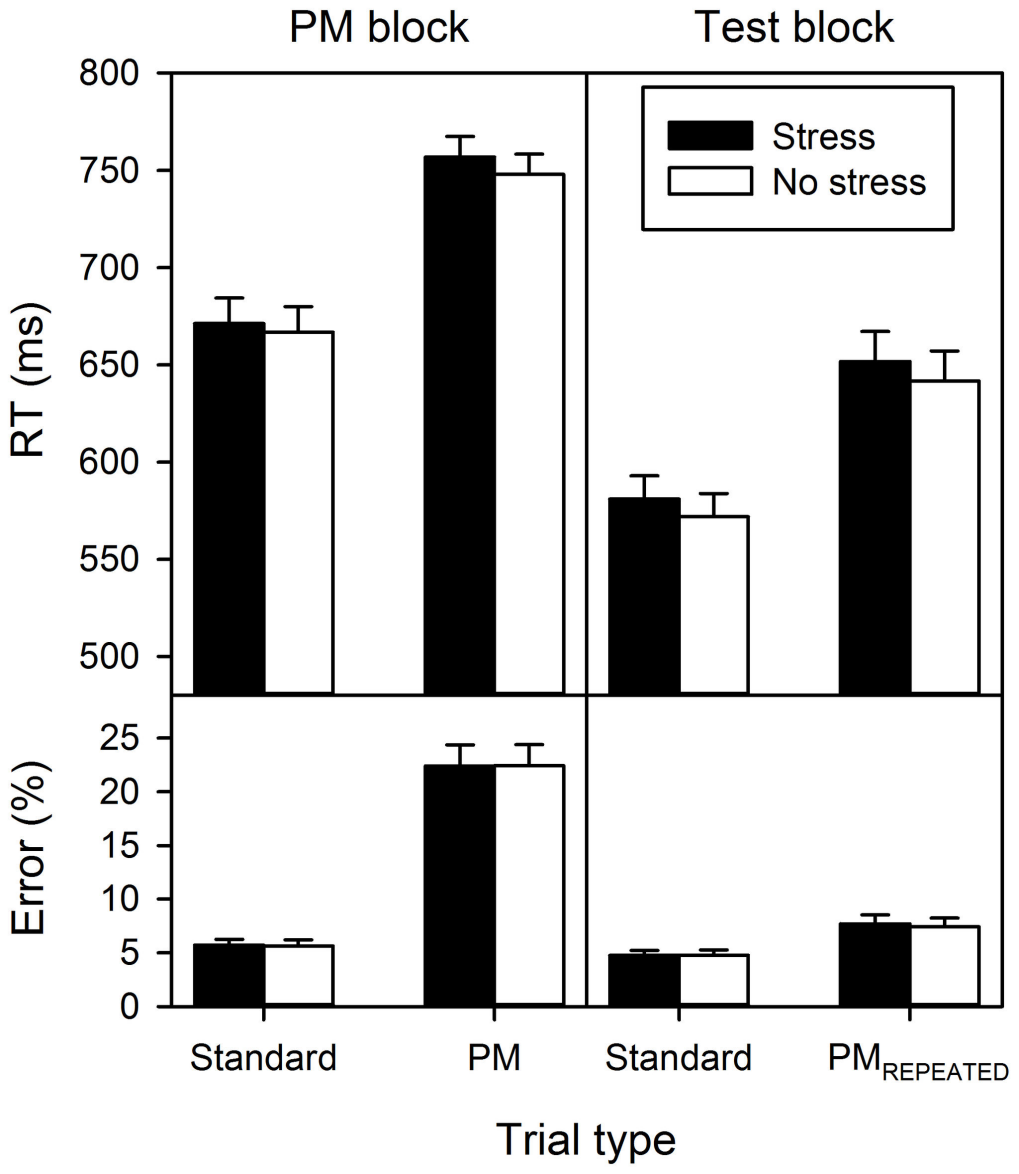
Cognitive performance. Mean response time (RT) and mean error rates for the Prospective memory (PM) block and the Test block as a function of trial type (PM block: standard vs. PM; Test block: standard vs. PM_REPEATED_) and treatment (stress vs. no stress). Error bars represent standard errors.

 Participants responded slower on PM trials (*M* = 752 ms) than on standard trials (*M* = 669 ms), *F*(1, 78) = 133.96, *p* < .001, η^2^ = .63. Mean RTs and differences between PM trials and standard trials did not vary across cognitive testing parts, *F*s < 1. Most importantly, mean RTs and differences between PM trials and standard trials were similar in stressed and non-stressed participants, *F*s < 1 (Figure 4, Table 1). Further, males (*M* = 730 ms) responded slower than females (*M* = 691 ms), *F*(1, 78) = 6.47, *p* = .013, η^2^ = .08. Descriptively, this difference tended to be more pronounced on standard trials (males: *M* = 695 ms, females: *M* = 643 ms) than on PM trials (males: *M* = 765 ms, females: *M* = 740 ms). However, the corresponding Trial type × Sex interaction missed significance, *F*(1, 78) = 3.27, *p* = .075, η^2^ = .04 (all further ps >.116).

**Table 1:** Mean response Times (in ms) for the prospective memory (PM) Block and the Test Block by Trial type (PM Block: Standard, PM; Test Block: Standard, PM_REPEATED_) and treatment (stress, No stress), and with the additional factors sex (female, male) and Time (Part 1 to Part 4).

		**Female**					**Male**					**Overall**
		Part 1	Part 2	Part 3	Part 4		Part 1	Part 2	Part 3	Part 4		
**Stress** (female: *n* = 21, male: *n* = 20)												
PM block	Standard	647 (49)	638 (54)	635 (45)	635 (44)		699 (101)	708 (122)	706 (134)	702 (124)		670 (92)
	PM	744 (64)	728 (42)	704 (58)	730 (70)		785 (86)	790 (94)	795 (97)	778 (83)		755 (67)
Test block	Standard	553 (51)	548 (41)	546 (37)	545 (40)		610 (89)	606 (104)	616 (133)	624 (126)		580 (87)
	PM_REPEATED_	624 (88)	609 (97)	623 (78)	602 (73)		693 (125)	672 (118)	681 (153)	710 (165)		651 (103)
**No stress** (female: *n* = 20, male: *n* = 21)												
PM block	Standard	654 (62)	644 (62)	649 (70)	644 (58)		682 (85)	690 (109)	694 (107)	677 (99)		667 (82)
	PM	755 (86)	757 (81)	750 (74)	748 (91)		748 (104)	749 (83)	726 (89)	750 (90)		748 (73)
Test block	Standard	553 (51)	550 (52)	551 (49)	551 (48)		594 (79)	594 (94)	595 (109)	586 (86)		572 (74)
	PM_REPEATED_	611 (79)	601 (74)	611 (74)	618 (101)		672 (126)	696 (155)	666 (142)	657 (122)		643 (104)

Standard deviations in parentheses.

 Participants committed more errors on PM trials (*M* = 22.4%) than on standard trials (*M* = 5.7%), *F*(1, 78) = 212.55, *p* < .001, η^2^ = .73 (Figure 4, Table 2). Trial type and time interacted, *F*(3, 234) = 5.74, *p* = .001, η^2^ = .07, with a steep initial decline of the difference in the error rates between PM trials and standard trials PM errors from the first (20.3%) to the second time point (13.9%), *F*(1, 78) = 13.06, *p* = .001, η^2^ = .14 (note: time part 3 and 4 remained at 16.4% each). Error rates did not differ between treatment groups, *F* < 1, or sexes, *F* < 1 (all further ps > .099).

**Table 2:** Mean Error Rates (in %) for the PM Block and the Test Block by Trial Type (PM Block: Standard, PM; Test Block: Standard, PM_REPEATED_) and Treatment (Stress, No Stress), and with the Additional Factors Sex (Female, Male) and Time (Part 1 to Part 4).

		**Female**					**Male**					**Overall**
		Part 1	Part 2	Part 3	Part 4		Part 1	Part 2	Part 3	Part 4		
**Stress** (female: *n* = 21, male: *n* = 20)												
PM block	Standard	5.5 (4.1)	6.1 (3.8)	6.4 (4.0)	7.4 (4.6)		4.3 (2.6)	5.3 (4.1)	5.3 (4.4)	5.3 (4.6)		5.7 (3.7)
	PM	29.5 (19.3)	20.5 (11.9)	20.2 (13.7)	26.8 (12.0)		22.5 (18.0)	20.6 (12.8)	20.0 (15.0)	19.1 (14.1)		22.4 (11.3)
Test block	Standard	4.9 (3.1)	5.8 (3.8)	5.7 (3.3)	6.8 (4.7)		3.5 (2.7)	3.1 (2.6)	4.2 (3.5)	3.9 (2.5)		4.8 (3.0)
	PM_REPEATED_	7.7 (9.3)	9.8 (10.2)	9.8 (10.7)	9.5 (7.0)		5.0 (7.7)	6.3 (7.6)	7.8 (11.3)	5.6 (7.5)		7.7 (3.4)
**No Stress** (female: *n* = 20, male: *n* = 21)												
PM block	Standard	5.1 (3.4)	5.0 (3.5)	5.3 (3.6)	5.2 (4.7)		5.4 (3.5)	5.4 (3.5)	7.0 (3.9)	6.6 (4.6)		5.7 (3.4)
	PM	24.7 (16.9)	16.3 (9.8)	20.6 (15.2)	17.5 (12.9)		25.0 (18.1)	20.2 (16.4)	28.6 (17.5)	26.5 (19.6)		22.5 (13.9)
Test block	Standard	4.1 (2.6)	4.3 (3.6)	3.9 (2.9)	4.5 (4.1)		4.7 (3.3)	4.6 (3.2)	6.2 (4.4)	5.9 (4.4)		4.8 (3.2)
	PM_REPEATED_	5.0 (5.2)	5.6 (7.8)	8.1 (8.4)	8.8 (10.4)		8.3 (8.5)	8.3 (8.0)	7.1 (8.0)	8.0 (10.5)		7.4 (5.4)

Standard Deviations in Parentheses.

#### Test block

Slower responses on PM_REPEATED_ trials (*M* = 647 ms) compared to standard trials (*M* = 576 ms) indicate aftereffects of completed intentions, *F*(1, 78) = 176.60, *p* < .001, η^2^ = .69. Mean RTs and aftereffects did not vary across time, *F*s < 1, and most importantly, were similar between treatment groups, *F*s < 1. Analogous to the PM block, male participants (*M* = 642 ms) responded slower than female participants (*M* = 581 ms), *F*(1, 78) = 10.51, *p* = .002, η^2^ = .12 (further ps > .097).

Aftereffects of completed intentions were also observed in error data with more errors on PM_REPEATED_ trials (*M* = 7.6%) than on standard trials (*M* = 4.8%), *F*(1, 78) = 46.28, *p* < .001, η^2^ = .37. Mean error rates and aftereffects were similar across time, *F*(3, 234) = 1.65, *p* = .179, η^2^ = .02, and *F* < 1, respectively. Although no main effects of treatment and sex were found, *F*s < 1, both factors interacted, *F*(1, 78) = 4.69, *p* = .033, η^2^ = .06 (all further *F*s < 1). Subsequent testing, however, revealed that neither males nor females showed significant differences in error rates between treatment conditions, *F*(1, 39) = 2.31, *p* = .137, η^2^ = .06, and *F*(1, 39) = 2.39, *p* = .130, η^2^ = .06, respectively.

#### Ongoing-task performance across blocks

Evidence for monitoring-based intention retrieval [48] was indicated by higher standard trial RTs in the PM block (*M* = 669 ms) compared to the Test block (*M* = 576 ms), *F*(1, 78) = 923.48, *p* < .001, η^2^ = .92. Monitoring costs remained constant over the time course of the experiment, *F*(3, 234) = 1.14, *p* = .334, η^2^ = .01, and did not differ between sexes, *F* < 1. Most importantly, monitoring costs were not affected by treatment, *F* < 1 (further ps > .308).

 Monitoring costs were also present in terms of increased error rates in the PM block (*M* = 5.7%) compared to the Test block (*M* = 4.8%), *F*(1, 78) = 25.04, *p* < .001, η^2^ = .24. Mean error rates on standard trials increased over the time course of the experiment (part 1: *M* = 4.7%, part 2: *M* = 5.0%, part 3: *M* = 5.5%, part 4: *M* = 5.7%), *F*(1, 78) = 18.01, *p* < .001, η^2^ = .19 (linear contrast), while monitoring costs did not, *F* < 1. Further, monitoring costs were neither affected by treatment nor by sex, *F*s < 1. The Treatment × Sex interaction missed significance, *F*(1, 78) = 3.94, *p* = .051, η^2^ = .05 (see Table 2, further ps > .111, η^2^ ≤ .03). 

Given that stress effects on cortisol tended to be most pronounced directly after stressor cessation, we conducted all analyses of cognitive performance again using only the first testing part after stress or no-stress treatment. Importantly, however, for both the PM block and the Test block, mean RTs and error rates as well as differences between standard and PM trials, and standard and PM_REPEATED_ trials, respectively, did not differ, *F*s < 1. Additionally, ongoing-task performance as well as monitoring costs did not differ between treatment groups, *F*s < 1.

## Discussion

In the current study, we investigated intention retrieval and deactivation following an acute psychosocial stressor (TSST) within the time period of stress-related elevated cortisol levels. We hypothesized that stress induction would either lead to decrements in PM performance and intention deactivation mediated by impaired PFC processing or to a processing-strategy shift such as increased PM performance at the cost of ongoing-task performance deterioration.

Results were rather clear: First, stress induction was successful in participants exposed to the TSST, as shown by a valid biological stress response (e.g., increased salivary cortisol) as well as immediate subjectively worse mood and increased restlessness compared to participants that were assigned to the no-stress control condition. Similar to previous studies, subjective consequences of TSST exposure died away relatively fast after cessation of the stressor, whereas neuroendocrine responses to stress were still present after 50 minutes [20,41]. Second, cognitive results also showed the predicted outcome: PM accuracy was within the normal range for PM studies (i.e., no ceiling effect occurred) and RTs and error rates were increased for PM_REPEATED_ trials compared to standard trials, indicating aftereffects of completed intentions [3,4]. Most strikingly, however, despite a strong biological and subjective stress response and standard effects in the cognitive task, stress did not exert *any* influence on PM performance, ongoing-task performance, and aftereffects of completed intentions.

The finding of completely preserved cognitive performance in the given task under conditions of acute psychosocial stress is remarkable, given the many precautions that were taken to detect any present interactions: Firstly, as a fundamental and important difference to Nater et al. [19], in the present study we controlled for unspecific performance criterion shifts assessing *both* RTs and accuracy. Even though, interpretations of null-findings have to be handled with care, we nevertheless believe that explanations with respect to insufficient statistical power are rather unlikely, as the present study with its relatively large number of participants and repeated-cycles design (i.e., high number of PM measurement time-points) was highly susceptible for even subtle stress induced performance differences.

Second, deliberately testing half females and males in both treatment groups enabled us to test for possible gender-effects [38,39]. Our results extend previous findings by demonstrating that preserved PM performance under stress is not confined to a gender-specific subsample (i.e., men) [19], but seems to reflect a general gender-unspecific phenomenon. 

Finally, we tested for putative time effects of stress on cognitive performances [20,40] by tracing cognitive performance during a long time interval after stressor cessation. Ongoing-task errors rates slightly increased within the time-course of cognitive testing, presumably due to increased fatigue as reported by the participants. In addition we found increased error rate differences between standard and PM trials especially in the first (compared to second) part after treatment. This finding suggests that although participants had extensively trained the PM task beforehand, it could not yet considered being habitual. Most importantly, these changes were similarly evident in both the stress and the no-stress group.

Findings from the present study contrast assumptions of stress-induced depletion of cognitive resources [34] and deterioration of PFC-related higher-cognitive functions [16], which were most probability required for performing the present PM task, as revealed by monitoring costs in terms of ongoing-task performance decrements in the PM block compared to Test block [17,48]. Alternatively to a general stress-induced impairment, we neither found evidence for the alternative assumption of resource re-allocation, for example, as a shift in processing strategy in conditions of stress [21], as has been found in terms of higher time-based PM hit rate at the cost of an increased number of clock checks [19]. However, the stress group in the present study performed equally well in the PM task as the no-stress group, without any strategic shifts such as adopting a rather spontaneous instead of monitoring-based retrieval mode [48,49], which was reflected in similar monitoring costs in both treatment groups.

Although the present work was especially designed to enable the detection of even subtle effects on PM and intention deactivation, a lot of work is still ahead of the scientific community and several questions remain open on the effects of stress on PM. For instance, it is conceivable that, although PM retrieval in the current experiment most likely relied on prefrontal cortex mediated monitoring (as reflected in monitoring costs) [17], the present PM task might not have been sufficiently demanding to require a shift in processing mode in conditions of acute psychosocial stress [21]. Adoption of such kind of even stronger resource-demanding monitoring based retrieval mode could be accomplished by using PM cues which are not only non-salient but also non-focal [49]. Additionally, more complex PM tasks [52] requiring maintenance of more sophisticated intended-action plans might be stronger affected by acute stress compared to the rather simple intended action (i.e., pressing the spacebar) applied in the present experiment.

 Similarly, stress did not affect intention deactivation in the current study. Nevertheless, it remains an open question whether this would also be the case in conditions requiring even stronger inhibitory processes to prevent interference with the ongoing task such as salient PM_REPEATED_ trials [2]. It is conceivable that when individuals with impaired executive functions (e.g., elderly) are exposed to salient PM_REPEATED_ trials under acute stress, the probability of commission errors increases resulting in intention deactivation failures. 

 Finally, findings from research on stress effects on episodic-long term memory [53] suggest that some stages (e.g., encoding) of intention memory might be less prone to stress effects than others (e.g., retrieval), or that stress might exert even opposing effects on different stages. Similarly, further research needs to determine whether specific components such as maintaining a complex intended-action plan might be affected by stress, whereas other components such as maintaining the PM cue might not. 

## Conclusions

In summary, although we found strong effects of stress on biological parameters and subjective mood as well as the expected cognitive data pattern, PM performance and intention deactivation were fully preserved under conditions of an acute psychosocial stressor. These findings are of crucial importance as they indicate that functioning of these cognitive abilities, which are essential for every-day life, seem to be quite reliable even under conditions of strong physiological alterations. However, at the same time, we acknowledge that the performance quality of prospective memory under stress might well depend on the task complexity and cognitive demands of the to-be-remembered intended action. 
